# Adherence to interdisciplinary tumor board recommendations as an expression of quality-assured patient care: results of a bicentric German analysis

**DOI:** 10.1007/s00432-023-05253-5

**Published:** 2023-08-17

**Authors:** Friederike Braulke, Kathrin Kober, Stefan Rieken, Tonia Brand, Tobias Hartz, Stefanie Seipke, Thomas Asendorf, Jörg Haier

**Affiliations:** 1 Comprehensive Cancer Center, University Medical Center Göttingen, von-Bar-Str. 2/4, 37075 Göttingen, Germany; 2 Department of Radiation Therapy and Radiooncology, University Medical Center Göttingen, Göttingen, Germany; 3 Klinisches Krebsregister Niedersachsen, Hannover, Germany; 4 Comprehensive Cancer Center Hannover, Hannover Medical School, Hannover, Germany; 5 Institute of Medical Statistics, University Medical Center Göttingen, Göttingen, Germany

**Keywords:** Cancer, Adherence, Tumor board, Quality management, Certification

## Abstract

**Purpose:**

Interdisciplinary tumor boards (ITBs) represent a central part of standard cancer care defining a guidelines-guided treatment plan adapted to the patient’s capabilities, comorbidities and wishes in a multi-professional team. The implementation rate of ITB recommendations can be monitored by structured adherence analyses. But (inter)national definitions how to measure the level of implementation are missing. Here, we present results of 4 years of ITB adherence analyses in a bicentric German Comprehensive Cancer Center (CCC).

**Methods:**

Between 2018 and 2021, for at least 1 month, the implementation rate of recommendations of 8 different ITBs of 2 CCC sites was evaluated manually according to harmonized criteria between both sites regarding the degree of implementation of ITB’s recommendations.

**Results:**

In total, 1104 cases were analyzed (65% male, 35% female). Mean distance from patient’s home to the CCC was 57 km (range 0.8–560.6 km). For 949 cases (86%) with known follow-up, the adherence rate was 91.9% (95% CI 0.9; 0.935). In 8.1%, ITB decisions were not implemented due to medical reasons (45.4%), patient’s wish (35.1%) and unknown reasons (19.5%). Logistic regression revealed neither age (OR = 0.998, *p* = 0.90), nor gender (OR = 0.98, *p* = 0.92) or the distance from patient’s home to the CCC (OR = 1.001, *p* = 0.54) were significantly associated with ITB adherence.

**Conclusion:**

ITB adherences analyses can serve as a quality management tool to monitor the implementation rate of ITB recommendations and to stay in contact with practitioners, other hospitals and state cancer registries to share data and resources in accordance with data protection requirements for continuously improvement of quality management and patient care.

**Supplementary Information:**

The online version contains supplementary material available at 10.1007/s00432-023-05253-5.

## Introduction

Every year, almost half a million people in Germany are newly diagnosed with cancer (Krebs in Deutschland für 2015/2016). With demographic changes, the incidence is expected to increase up to further 20% until 2030 (Krebs in Deutschland für 2015/2016). Rapid scientific progress in all areas of oncology leads to an increasing complexity and individualization of diagnostic and therapeutic options for cancer patients. One goal of the National Cancer Plan is to design transparent evidence-based structures in Germany that ensure competent, cross-sectoral quality-assured care of all cancer patients (German Ministry of Health. National Cancer Plan. Berlin 2012). In order to issue an optimal treatment plan guided by clinical practice guidelines and adapted to each individual cancer patient including the patient’s capabilities, comorbidities and wishes, interdisciplinary tumor boards (ITBs) now represent a central part of the standard cancer care in Germany. Specialists in surgery, pathology, radiology, radiooncology and medical oncology, as well as experts from other disciplines discuss each individual patient in regular meetings to define a recommendation according to national and international clinical practice guidelines and patient’s wishes. In organ-specific cancer centers certified according to the criteria of the German Cancer Society (Deutsche Krebsgesellschaft, DKG), all cancer patients have to be presented to ITBs at least at the time of initial diagnosis, recurrence or disease progression, and before and after surgical intervention ((Kowalski et al., 2017; Guideline Program in Oncology 2021; Deutsche Krebsgesellschaft; Catalogue of requirements for Organ Cancer Centers; Griesshammer and Wesselmann 2019; Catalogue of requirements for breast cancer centers 2022). In large certified cancer centers, there are ITBs for each tumor entity available that take place at least once a week as virtual or physical meetings. In addition, the DKG-certified organ-specific cancer centers and oncology centers are obliged to record and monitor the adherence to ITB’s recommendation (Catalogue of requirements for Oncology Centers 2022). For certified skin cancer centers and pediatric cancer centers, there is even a separate indicator that has to be evaluated every year as a requirement for certification: deviation from ITB’s recommendation should not exceed 25% in skin cancer centers (Data Sheet Skin Cancer Centers 2022) and less than 5% in pediatric cancer centers (Catalogue of requirements for pediatric cancer centers 2022).

There might be different reasons for a deviation from the tumor board’s recommendation:

- For medical reasons, there might be unpredictable events (e.g. stroke, heart attack) that make it impossible to implement ITB’s recommendation.
- The patient may refuse the recommended diagnostic or therapy.
- The ITB’s recommendation might be too complex or unrealistic to implement for the treating physician (e.g. because the patient’s general condition or concomitant diseases are not known to the ITB and do not allow ambitious therapies, or innovative treatment options that are not available in rural regions far away from the cancer center).
- The treating practitioners go beyond ITB’s recommendation and treat the patients at their own discretion.

In order to prove quality-assured cancer care, ITB adherence analyses should be performed regularly in certified cancer centers. For this purpose, the center needs complete information on the course of the disease of the patients (e.g. type and duration of therapy, treatment response, recurrence, survival status). If the patient is still treated at the cancer center after ITB, all information are available. If the patient receives further treatment close at home in distant institutions, follow-up information might get lost. The treating physician can provide information on the further course of the disease to the center, or the cancer center holds additional staff to actively enquire and obtain the follow-up data, for example by calling the treating practitioners in regular intervals. Both ways are cost-intensive and personnel-intensive. On the other hand, all medical physicians and dentists in Germany are legally obliged to report e.g. cancer diagnoses, start and end of therapy, changes in the course of the disease, recurrences, metastasis, secondary malignancies, results of follow-up care and the patient’s death to their respective state cancer registry in accordance with legislation (§65c SGB V, Table 1) (Sozialgesetzbuch (SGB V), 2022). But events for reporting and inconspicuous follow-up examinations have not been completely identically defined and harmonized between the federal states in Germany, as yet (Table 1).

**Table 1:** Events for reporting to state cancer registries for all medical physicians and dentists in Germany

Events for reporting to state cancer registries for all medical physicians and dentists in Germany
Cancer diagnosis
Histopathological, cytological, molecular information or autopsy data
Start and ending of treatment
Changes in the course of the disease relevant for treatment (progression, relapse, metastasis)
Follow-up information
Death

Adopted to Klinisches Krebsregister Niedersachsen, Hannover, Germany https://www.kk-n.de/melder-aerzte/meldepflicht/and Sozialgesetzbuch (SGB V), Fünftes Buch, Gesetzliche Krankenversicherung: § 65c SGB V Klinische Krebsregister, Stand: Zuletzt geändert durch Art. 1b G v. 20.12.2022 I 2793 (https://www.sozialgesetzbuch-sgb.de/sgbv/65c.html20.05.2023).

In accordance with the National Cancer Plan, which was launched by the Federal Ministry of Health together with the German Cancer Society (DKG), the German Cancer Aid (Deutsche Krebshilfe, DKH) and the Association of German Cancer Centers (Arbeitsgemeinschaft Deutscher Tumorzentren, ADT) (German Ministry of Health. 2012), the state cancer registries in Germany should record the quality of cancer care, assure standardized documentation and report quality data back to health care providers and general public.

In concerns of analyzing the adherence to ITB’s recommendation, there are no nationally or internationally harmonized definitions available how to measure the level of implementation (e.g. treatment modality, different drugs, different modalities, multimodal treatment plans and drug combinations, different dose and time schedules).

Here, we present the results of 4 years of ITB adherence analyses in a bicentric Comprehensive Cancer Center of excellence (CCC) and discuss the challenges and chances for hospitals and health care providers for closer cooperation with the responsible state cancer registry.

## Methods

Between 2018 and 2021, for eight different multi-professional ITBs of the two German CCC sites, the implementation rate of recommendations was investigated for at least 1 month. At both sites, the ITBs of the skin cancer centers, the lung cancer centers, the sarcoma centers including musculo-skeletal malignancies, the urogenital cancer centers, and the gastrointestinal cancer (GI) centers were analyzed at least once during the observation time. Additionally, the ITBs of the center of hematological malignancies, the head–neck cancer center and the neuro-oncological center were analyzed once at one site. Rare or very complex cases at both sites were discussed in the ITB most suitable at anatomical site. At both sites, ITBs took place as physical meetings, recommendations were recorded within the joint regular tumor documentation system Onkostar (IT-Choice, Karlsruhe Germany). All physicians had personal access to the tumor documentation system and were able to register patients to the ITB (Braulke et al. 2023). In accordance with certification requirements senior physicians of surgery, pathology, radiology, radiooncology and medical oncology, and specialists of other disciplines have to be present in all ITBs, thus the composition of ITB participants is comparable in all boards at both sites. However, the requirements slightly differ between the different site-specific types of ITB. For each patient presented and discussed in an ITB, there is a written recommendation of the ITB. According to certification requirements and institutional standard operating procedures (SOPs) of the CCC sites, patients have to be discussed again e.g. after surgery or in case of new information or unexpected events.

The evaluation was performed manually according to harmonized criteria between both CCC sites:

- Surgical intervention yes/no
- Radiation therapy yes/no
- Systemic therapy yes/no
- Local procedures yes/no
- Aftercare/watch and wait/best supportive care yes/no
- Other diagnostics yes/no

Different drugs, dosages, the type of surgery or individual radiation protocols were not considered for this analysis. If treatment was started according to the ITB recommendation, it was considered as adherence (intention to treat according to protocol). The complexity of ITB recommendations depends on the medical case. For each patient, the kind of treatment or intervention implemented after ITB was documented during the course of the disease. The final assessment was defined as “compliant”, “deviating” or “unknown” if the patient’s further cancer care (see examples, Table 1 supplementary information)

1. Was in line with ITB recommendation (compliant),
2. Differed from ITB recommendation regarding the therapy modalities recommended: surgery, radiotherapy, systemic therapy, local therapy, supportive therapy, further diagnostics (deviating),
3. Was not known to the cancer center (unknown).

In case of divergence of treatment and ITB recommendation, the reasons for deviation were analyzed and clustered into 3 groups:

- Patient’s wish
- Deterioration of the patient’s general condition or other medical reasons that no longer allowed implementation
- Unknown reason.

The individual documentation was carried out by the educated staff for tumor documentation, medical control and evaluation were performed centrally by the quality management and the leading physicians of the certified oncology centers of both sites (FB, JH). The results were made available to the relevant organ-specific cancer centers, discussed with the respective ITB chairs and teams and presented in local and regional quality conferences.

## Statistics

Generalized linear models (logistic regression) were employed to analyze associations between age, sex, tumor board and distance on adherence. Proportions were reported with logit-transformed 95%-confidence intervals (95% CI) using estimated marginal means (Lenth 2022) and odds ratios (OR) tested against ${H}_{0}:\mathrm{OR}=1$ at two-sided significance level of 5%. All analyses were performed in R version 4.2.0 (R Core Team 2022).

## Results

In total, 1104 cases from 8 different organ-specific ITBs were analyzed at 2 sites. Table 2 gives the patient cohort examined: there were 65% male and 35% female cases with a mean age of 64.1 years (range 6.1–97.6 years). The cancer sites reflect the ITBs analyzed: patients with GI cancer were most often discussed, followed by urogenital cancer, musculo-skeletal tumors including sarcoma, skin cancer and lung cancer patients. The mean distance from patient’s home to the responsible CCC was 57.8 kilometers (km, standard deviation (SD) 57.4 km, range 0.8–560.6 km).

**Table 2:** Characteristics of cases discussed in ITBs (*n* = 1104 cases)

	CCC A	CCC B	Total
Number of cases discussed, *n* (%)	646 (59)	458 (41)	1104 (100)
Mean age, years (range)	64.3 (16.2–97.6)	63.7 (6.1–94.2)	63.9 (6.1–97.6)
Gender, *n* (%)			786/455 (63/37)
Male	430 (66.5)	284 (62.0)	706 (64.4)
Female	217 (33.5)	174 (38.0)	391 (35.6)
Cancer diagnoses, *n* (%)			
Gastrointestinal cancer	260 (40.2)	95 (20.7)	355 (32.2)
Urogenital cancer	71 (11)	67 (14.6)	138 (12.5)
Sarcoma and musculo-skeletal tumors	16 (2.4)	97 (21.1)	113 (10.2)
Skin cancer	30 (4.6)	79 (17.2)	109 (9.9)
Lung cancer	47 (7.3)	61 (13.3)	108 (8.7)
Hematological malignancies	54 (8.3)	3 (0.6)	57 (5.1)
Head-and-neck cancer	46 (7.1)	7 (1.5)	53 (4.8)
Neuro-oncological diseases	28 (4.3)	2 (0.4)	30 (2.7)
Gyneco-oncological diseases	5 (0.8)	9 (2)	14 (1.3)
Endocrine malignancies	0 (0)	2 (0.4)	2 (0.2)
Other malignancies	89 (13.8)	36 (8)	125 (11.3)
Mean distance to the CCC, km (range)	58.0 (2.5–431.8)	57.3 (0.8–560.6)	57.8 (0.8–560.6)

*ITB* interdisciplinary tumor boards, *CCC* Comprehensive Cancer Center, *Mean distance to the responsible CCC* The mean distance from patient’s home to the responsible CCC, *km* kilometers

**Fig. 1:**
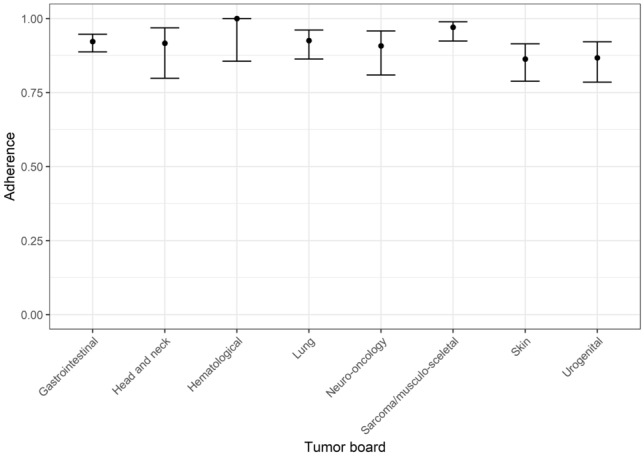
Adherence rate to tumor board’s recommendations

In total, for 949 cases (86%), follow-up data were available. The overall adherence rate to ITB recommendations was 91.9% (95% CI 0.9; 0.935) ranging from 86.3 to 100% for individual tumor boards (see Table 3, Fig. 1).

**Table 3:** Adherence to interdisciplinary tumor board recommendations (*n* = 949 cases with known follow-up)

Tumor board	CCC A		CCC B		Total	
	Cases, *n* (%)	Adherence, % (95% CI)	Cases, *n* (%)	Adherence, % (95% CI)	Cases, *n* (%)	Adherence, % (95% CI)
GI cancer	250 (45.0)	92.8 (0.89; 0.95)	71 (17.9)	90.1 (0.81; 0.95)	321 (34.0)	92.2 (0.89; 0.95)
Urogenital cancer	50 (9.0)	96.0 (0.85; 0.99)	48 (12.1)	77.1 (0.63; 0.87)	98 (10.3)	86.7 (0.78; 0.92)
Skin cancer	29 (5.2)	82.8 (0.65; 0.93)	88 (22.2)	87.5 (0.79; 0.93)	117 (12.3)	86.3 (0.79; 0.91)
Sarcoma and musculo-skeletal tumors	14 (2.5)	92.9 (0.63; 0.99)	121 (30.1)	97.5 (0.93; 0.99)	135 (14.2)	97.0 (0.92; 0.99)
Lung cancer	53 (9.6)	98.1 (0.88; 0.99)	68 (17.2)	88.2 (0.78; 0.94)	121 (12.8)	92.6 (0.86; 0.96)
Hematological malignancies	44 (8.0)	100.0 (0.86; 1.00)	na	na	44 (4.6)	100.0 (0.86; 1.00)
Head-and-neck cancer	48 (8.7)	91.7 (0.80; 0.97)	na	na	48 (5.0)	91.7 (0.80; 0.97)
Neuro-oncological diseases	65 (11.8)	90.8 (0.81; 0.96)	na	na	65 (6.8)	90.7 (0.81; 0.96)
Total	553 (100)	93.3 (0.91; 0.95)	396 (100)	90.0 (0.87;0.93)	949 (100)	91.9 (0.9; 0.93)

*GI* gastrointestinal cancer, *CCC* Comprehensive Cancer Center, *95% CI* 95%-Confidence interval, *na* not analyzed

**Fig. 2:**
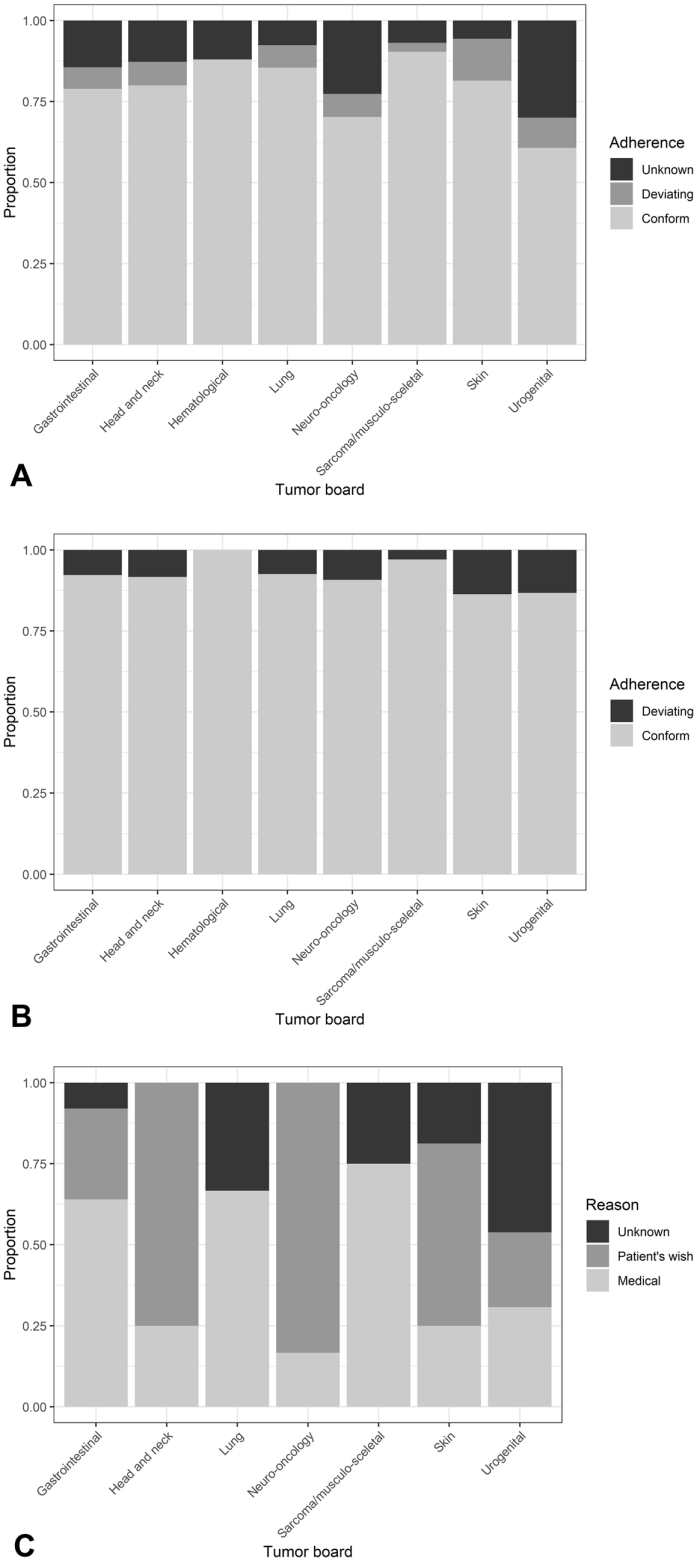
**A** Adherence to tumor board’s recommendations including all cases analyzed (*n* = 1104): Proportion of conformance, deviation and unknown follow-up for tumor boards for gastrointestinal cancer (*n* = 375), head and neck cancer (*n* = 55), hematological malignancies (*n* = 50), lung cancer (*n* = 131), neuro-oncology (*n* = 84), sarcoma and musculo-skeletal tumors (*n* = 145), skin cancer (*n* = 124) and urogenital cancer (*n* = 140). **B** Adherence to tumor board’s recommendations including all cases with known follow-up data (*n* = 949): Proportion of conformance and deviation for tumor boards for gastrointestinal cancer (*n* = 321), head and neck cancer (*n* = 48), hematological malignancies (*n* = 44), lung cancer (*n* = 121), neuro-oncology (*n* = 65), sarcoma and musculo-skeletal tumors (*n* = 135), skin cancer (*n* = 117) and urogenital cancer (*n* = 98). **C** Reasons for deviation (*n* = 77 cases): Medical reasons, patient’s wish or unknown reasons for tumor boards for gastrointestinal cancer (*n* = 25), head and neck cancer (*n* = 4), hematological malignancies (*n* = 0), lung cancer (*n* = 9), sarcoma and musculo-skeletal tumors (*n* = 4), skin cancer (*n* = 16) and urogenital cancer (*n* = 13)

There was no significant difference between the two sites (*p* = 0.0594) with adherence rates of 0.90 and 0.93 on the individual sites (Table 3). In 8.1% (77/949) of cases, further treatment differed from the tumor board recommendation, mostly due to medical reasons (45.4%), followed by patient’s wish (35.1%) and other or unknown reasons (19.5%). In 14.0% (155/1104) of cases, there was no information on further course of the disease or therapy (Fig. 2A–C).

**Fig. 3:**
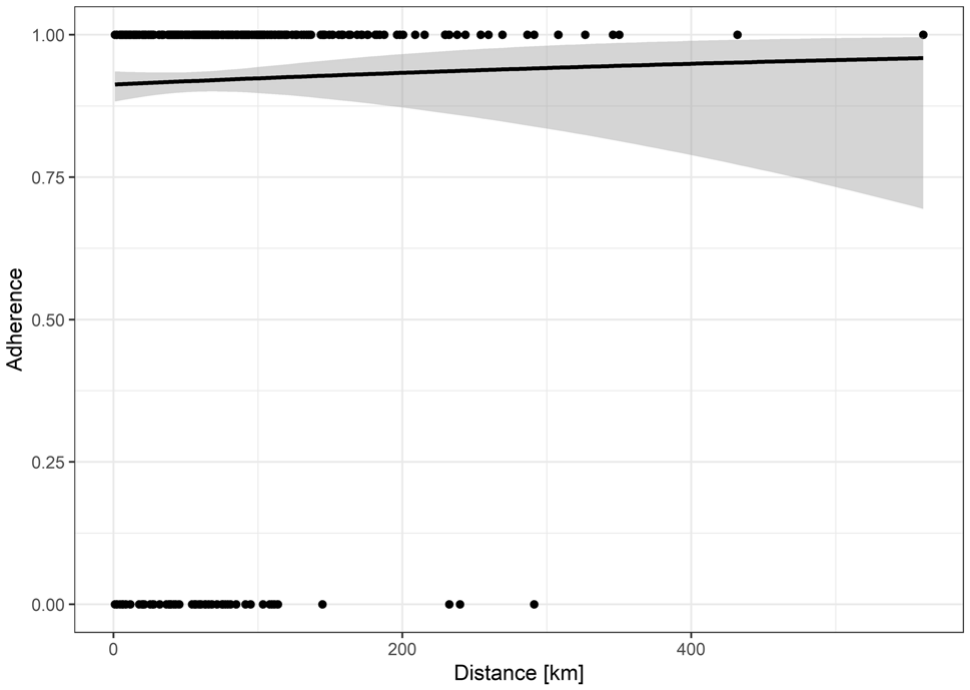
Logistic regression for the distance from patient’s home to the cancer center (in kilometers (km): No significant impact of distance from patient’s home to the cancer center on tumor board adherence (OR = 1.001, *p* = 0.54)

Logistic regression revealed neither age in years (OR = 0.998, *p* = 0.90) or gender (OR = 0.98, *p* = 0.92) showed a significant impact on adherence to ITB implementation rate (Table 2). Even the distance from patient’s home to the cancer center in km had no significant impact on ITB adherence (OR = 1.001, *p* = 0.54, Table 2, Fig. 3).

## Discussion

Multi-professional recommendations of interdisciplinary tumor boards of certified organ-specific cancer centers should be driven by national and international clinical practice guidelines and consider patient’s comorbidities and wishes to offer an optimal treatment to each patient (Blazeby et al. 2006; Lamb et al. 2011; Soukup et al. 2018; Griesshammer and Wesselmann 2019).

The results of this bicentric systematic analysis of ITB adherence over a period of 4 years showed that the implementation rates of ITB’s recommendations are stable over the years (average > 90%) and show no significant differences between the two sites. The main causes for deviation from ITB’s recommendation were medical reasons and patient’s wish. Therefore, harmonized SOPs and homogeneous requirements for certification procedures determine that patients are presented and discussed again in the ITB in case of unexpected medical events that make the implementation of the former ITB recommendation impossible. Our data underline the importance of knowing the patient’s wish at the time of first ITB presentation. Interestingly, the distance from patient’s home to the cancer center was not significantly associated with the implementation of ITB recommendation, although some patients live up to 560 km far away from the cancer center. This stresses the need of good communication and participation of treating practitioners and other physicians in hospitals away from the cancer center.

Optimal preparation of the ITB presentations on one hand and the complete knowledge of tumor parameters as well as the patient’s wish, concomitant diseases, the home care setting, the place of following treatment and other individual factors of the patient on the other hand are essential for the multi-professional discussions during the ITB and influence the quality of decision-making (Wood et al. 2008; Lamb et al. 2011; Soukup et al. 2018; Braulke et al. 2023).

As yet, there are only few published data on tumor board adherence available (Petty and Vetto 2002; Lutterbach et al. 2005; Blazeby et al. 2006; Bumm et al. 2007; Leo et al. 2007; Wood et al. 2008; Lamb et al. 2011; Basta et al. 2017; Freytag et al. 2020). In most cases, the concordance of tumor board recommendation and treatment afterward was defined as ITB adherence (Petty and Vetto 2002; Lutterbach et al. 2005; Blazeby et al. 2006; Bumm et al. 2007; Leo et al. 2007; Wood et al. 2008; Lamb et al. 2011; Basta et al. 2017; Freytag et al. 2020). The focus was set on the “therapy columns” surgery, chemotherapy, radiotherapy, or supportive care and not separated for e.g. different drugs or dosages, the type of surgery or radiation schedules. In a prospective study from the United Kingdom (Blazeby et al. 2006), 271 ITB decisions were examined in patients with GI cancer. The authors described an implementation rate of 84.9% (230/271), 15.1% were not implemented. The reason for deviations were comorbidities (43.9%) and patient’s wish (34.2%). In 19.5% of cases, the decision was changed because more relevant clinical information was available after the ITB (Blazeby et al. 2006). Another prospective multicenter study in the United States of America showed an adherence rate of 84% in 153 ITB recommendations analyzed (Petty and Vetto 2002). A further British study examined 201 ITB decisions for patients with colorectal cancer prospectively and showed an implementation rate of 90% (Wood et al. 2008). As the main cause of non-adherence, the authors mentioned concomitant diseases, patient’s wish, new clinical information and unknown reasons (Wood et al. 2008). In a French analysis of a lung cancer board, a deviation rate of 4.4% was detected, mainly due to patient’s rejection of the therapeutic option recommended and reduced general condition of the patient (Leo et al. 2007). Two German studies showed ITB adherence rates of 96.03% in patients with esophageal and gastric cancer (Bumm et al. 2007) and 91% in brain cancer patients (Lutterbach et al. 2005). Since there are no harmonized national or international definitions of “tumor board adherence” we defined our own criteria how to measure adherence to ITB recommendation in a bicentric real-world CCC setting. Following published data, we focused on “therapy columns”, not on individual drugs or different procedures in surgery or radiooncology. Starting a treatment was considered as adherence comparable to intention to treat.

The repeated discussion of a patient during the course of the disease in ITBs can be associated with a significant survival advantage (Freytag et al. 2020). In case of relapse, progression or unexpected medical events (e.g. a stroke or a heart attack) or patient’s refusal that make an implementation of the ITB decision impossible, the patient should be discussed again within the ITB to evaluate the new situation again in a multi-professional team of experts and recommend a new, best matching, guidelines-guided therapeutic option. Previous studies for different cancer types have already shown that the interdisciplinary meetings in multi-professional tumor boards lead to improved overall survival of cancer patients (Bydder et al. 2009; Brar et al. 2014; Blay et al. 2017). First results of the recently published WiZen study (“Effectiveness of Care in Certified Cancer Centers in Germany,” ClinicalTrials.gov: NCT04334239; Wizen- Studie https://www.krebsgesellschaft.de/deutsche-krebsgesellschaft-wtrl/willkommen/presse/pressearchiv/wizen-projekt-bessere-ueberlebenschance-bei-krebsbehandlung-in-ze.html) confirm better survival rates of cancer patients treated in certified cancer centers with corresponding interdisciplinary tumor boards and evaluated structures (Roessler et al. 2022).

Compared to previously published national and international data, the large cohort analyzed here bicentrically shows a very good adherence rate (> 91%) regarding those patients with known follow-up data. Lutterbach and colleagues (2005) also reported ITB adherence analyses only for patients with known follow-up who had been treated at their own hospital. Leo and colleagues (2007) emphasized the additional staff that is necessary for the cancer center to obtain the information from following practitioners outside the cancer center about the patient’s further clinical course and treatment as well as response to therapy. Both CCCs of this analysis need additional personnel resources as well to collect follow-up data from treating physicians near at home to perform structured ITB adherence analyses as a harmonized tool of quality management and to fulfill certification requirements. And still, there were 14% of cases left with unknown follow-up. This is in line with former published data that a structured feedback system is needed to collect long-term follow-up data of all cancer patients to assure quality and further development of quality structures for better patient care (Homayounfar et al. 2014; Quero et al. 2020). ITB adherences analyses can serve as good-quality management tool of cancer centers to monitor the implementation rate of ITB recommendations and to stay in contact with treating practitioners, contracted partners in other hospitals and with the state cancer registries. State cancer registries collect and analyze all data of cancer patients for e.g. quality conferences, public health reporting and health care research (Robert-Koch-Institut, GeKiD (eds) 2019; Epidemiologisches Krebsregister Niedersachsen (EKN, Registerstelle), Klinisches Krebsregister Niedersachsen (KKN) und Klinische Landesauswertungsstelle Niedersachsen (KLast) (Hrgs) Im Auftrag des Niedersächsischen Ministeriums für Soziales, Gesundheit und Gleichstellung 2021). In addition to the medical history data, the state cancer registry also has information about a patient’s death or relocation from the official registration offices. Both the cancer centers and the state cancer registries should work closely together to share data and resources in accordance with data protection requirements to continuously improve quality management and cancer patient care.

## Limitations and strength

### Limitations

One limitation of this analysis is a potential bias caused by analyzing cases or recommendations, not individual patients. In line with certification requirements or institutional SOPs at the CCCs some patients (< 10%) needed to be presented in ITBs twice (e.g. before and after surgery), less than 1% was discussed three times. This analysis was focused on the adherence to the ITBs recommendation. For further studies individual patient’s history and course of the disease should be considered including dosages, types of surgery and radiation protocols. Another limitation might be missing information about the reasons for patient’s refusal of diagnostic or therapeutic steps recommended by the ITB. This should be considered in future trials. For comparison with other data published standardized international criteria about ITB adherence would be helpful.

### Strengths

The results of this bicentric analysis and involvement of a large variety of different tumor board types and cancer entities represent a real-life setting instead of a controlled clinical trial. With regard to limited resources of hospitals and outpatient units, the resources and opportunities of national and state cancer registries should be included in standardized workflows between the cancer center and the treating oncological care-givers.

## Conclusions

Structured adherence analyses by monitoring the implementation rates of ITB’s recommendations can serve as a quality management tool. National or state cancer registries can support physicians in cancer centers, general hospitals and in- and outpatient units by providing clinical follow-up data for improving outcome research and cancer care.

## Supplementary Information

Below is the link to the electronic supplementary material.

Supplementary file1 (DOCX 14 KB)

## Data Availability

The datasets analyzed during the current project are available from Friederike Braulke and Thomas Asendorf, University Medical Center Göttingen, Göttingen, Germany, on reasonable request.
