# Supplementary material for: Adherence to interdisciplinary tumor board recommendations as an expression of quality-assured patient care: results of a bicentric German analysis
Source: J Cancer Res Clin Oncol. 2023 Aug 17;149(16):14775–84. doi: 10.1007/s00432-023-05253-5 (PMC10602985; doi:10.1007/s00432-023-05253-5)
Supplement: Supplementary file 1 — Supplementary file1 (DOCX 14 KB) [file 432_2023_5253_MOESM1_ESM.docx]

Supplementary Information Table 1: Examples of tumor board recommendation, documentation and evaluation of ITB adherence.

| **ITB recommendation** | **Documentation of recommendation** | **Documentation of the course of the disease** | **Evaluation of ITB adherence** |
| --- | --- | --- | --- |
| Patient with adenocarcinoma of the pancreatic head, stenosis of the V. mes. sup., histologically proven metastasis in all liver segments and bone lesions of the spinal column without signs of instability. Port implantation and chemotherapy (FOLFOX) in palliative intention are recommended, bisphosphonate therapy is possible after dental check. | Surgery: No  Radiotherapy: No  Systemic (Chemo-)therapy: Yes  Local procedures: No  After Care/Best supportive care: No  Further diagnostics: No. | Surgery: No  Radiotherapy: No  Systemic (Chemo-)therapy: No  Local procedures: No  After Care/Best supportive care: No  Further diagnostics: No. | Deviating.  Reason for deviation:  Patient´s wish. |
| Patient with chronic lymphatic leukemia for many years now with progressive disease and abdominal bulky disease. A systemic treatment with Venetoclax/Obinutuzumab or oral therapy with acalabrutinib is recommended. | Surgery: No  Radiotherapy: No  Systemic (Chemo-)therapy: Yes  Local procedures: No  After Care/Best supportive care: No  Further diagnostics: No. | Surgery: No  Radiotherapy: No  Systemic (Chemo-)therapy: Yes  Local procedures: No  After Care/Best supportive care: No  Further diagnostics: No. | Conform. |
| Patient after radical prostatectomy. PSA-guided after care is recommended. | Surgery: No  Radiotherapy: No  Systemic (Chemo-)therapy: No  Local procedures: No  After Care/Best supportive care: Yes  Further diagnostics: No. | Surgery: Unknown  Radiotherapy: Unknown  Systemic (Chemo-)therapy: Unknown  Local procedures: Unknown  After Care/Best supportive care: Unknown  Further diagnostics: Unknown | Unknown. |

ITB: Interdisciplinary tumor board. FOLFOX: folinic acid, fluorouracil and oxaliplatin.
